# Melinjo-derived Gnetin C restores metabolic balance *via* dual adipose and hepatic effects in high-fat diet mice

**DOI:** 10.1038/s41598-025-25705-x

**Published:** 2025-11-25

**Authors:** Tomoki Kishimoto, Aoi Nasu, Mai Uemura, Keisuke Kawano, Choyo Ogasawara, Ayami Fukuyama, Hirofumi Nohara, Ryunosuke Nakashima, Noriki Takahashi, Yukio Fujiwara, Tomoki Ikuta, Mary Ann Suico, Hirofumi Kai, Tsuyoshi Shuto

**Affiliations:** 1 Department of Molecular Medicine Graduate School of Pharmaceutical Sciences, Kumamoto University, 5-1 Oe-Honmachi, Chuo-ku, Kumamoto, 862-0973 Japan; 2 Program for Fostering Innovators to Lead a Better Co-being Society, Kumamoto University, 2-39-1 Kurokami, Chuo-ku, Kumamoto, 862-8555 Japan; 3 Health Life Science S-HIGO Professional Fellowship Program Kumamoto University, 2-39-1 Kurokami, Chuo-ku, Kumamoto, 862-8555 Japan; 4 Department of Cell Pathology Graduate School of Medical Science, Kumamoto University, 1-1-1 Honjyo, Chuo-ku, Kumamoto, 860-8556 Japan; 5 Institute for Bee Products and Health Science Yamada Bee Company, Inc., 194 Ichiba, Tomata-gun, Kagamino-cho, 708-0393 Japan; 6 Global Center for Natural Resources Sciences Faculty of Life Sciences, Kumamoto University, 5-1 Oe-Honmachi, Chuo-ku, Kumamoto, 862-0973 Japan

**Keywords:** Obesity, Metabolic disorders

## Abstract

Multi-organ regulation underlies metabolic health, especially in the context of adipose-liver dysfunction during obesity. Previous findings identified Melinjo seed extract (MSE) as a promising modulator of metabolic disorders, although its active component remained unknown. Gnetin C, a *trans*-resveratrol dimer from MSE, likely serves as the key factor, yet its direct metabolic role remains unclear. Here, Gnetin C was administered to high-fat diet (HFD)-fed mice, which significantly improved body weight and fasting glucose, attributed to enhanced adiponectin (APN) multimerization. In adipose tissue, Gnetin C directly promotes APN multimerization and suppresses fat accumulation by up-regulating the PPARγ-DsbA-L axis, while concurrently modulating hepatic Sirt1, which may contribute to increased FGF21 production. This paracrine FGF21 signaling, suggested by elevated Fgfr1 in hepatocytes and βKlotho in adipocytes, further augments APN multimerization. These findings underscore the importance of a multi-tissue approach to obesity management and position Gnetin C as an integrative therapeutic candidate, restoring metabolic balance *via* dual adipose and hepatic effects in HFD mice.

**Supplementary Information:**

The online version contains supplementary material available at 10.1038/s41598-025-25705-x.

## Introduction

Type 2 diabetes, which accounts for roughly 90% of diabetes cases, arises from a combination of genetic predisposition and lifestyle factors such as poor diet and insufficient exercise^1^. As a chronic condition, diabetes demands both effective prevention strategies and mitigation of severe complications, including neuropathy, retinopathy, and nephropathy, which can significantly impact patients’ quality of life and healthcare systems^2^. The global increase in body mass index (BMI) underscores obesity as a critical risk factor for diabetes^3^. Although current therapies exist, their limited efficacy, elevated cost, and potential adverse effects often confine their use to the most severe cases, highlighting an urgent need for more effective and broadly accessible interventions^4^. Against this backdrop, the search for novel agents derived from natural products has gained momentum, driven by the unique biochemical mechanisms and favorable safety profiles that such compounds often exhibit^5^.

Resveratrol, a polyphenol in grape skins, has attracted substantial interest because of its anti-inflammatory, antioxidant, and anti-aging properties, and has been investigated in a range of disease contexts^6^. Several studies have documented Resveratrol’s positive effects on obesity and diabetes in high-fat diet (HFD)-fed mice^7^. In particular, resveratrol promotes the expression of disulfide bond A oxidoreductase-like protein (DsbA-L) in adipocytes, thereby enhancing the multimerization of adiponectin (APN), a process that alleviates metabolic dysfunction^8,9^. However, its rapid metabolism and low bioavailability present major obstacles to clinical application^6^. Consequently, Gnetin C, a *trans*-resveratrol dimer demonstrating greater in vivo stability^10–12^, emerged as a promising alternative.

Gnetin C is abundant in the seeds of Melinjo (*Gnetum gnemon L.*)^13^, a Southeast Asian plant often referred to locally as the “Tree of Life.” Melinjo seed extract (MSE) reportedly confers various health benefits, including anti-cancer, anti-periodontitis, anti-bacterial and anti-diabetic effects^13–17^, and has demonstrated high safety margins in toxicological evaluations (LD₅₀ > 5,000 mg/kg; NOAEL = 1,000 mg/kg)^18^. Prior work showed that oral MSE administration improves obesity and diabetes in HFD-fed mice through mechanisms involving DsbA-L and APN^19^, yet the key active component and its precise pharmacological mechanisms remain unclear.

Multi-organ regulation underlies metabolic health, especially in the context of adipose-liver dysfunction during obesity^20–22^. MSE influences both of these organs and was previously identified as a promising modulator of metabolic disorders, yet its active constituent was unknown. Gnetin C, a *trans*-resveratrol dimer found in MSE^13^, likely fulfills that role, but its direct metabolic function remains to be clarified. Consistently, our recent work suggests that Gnetin C ameliorates non-alcoholic fatty liver disease (NAFLD) in a manner similar to MSE^23^, implicating this compound as the prime candidate behind MSE’s multi-organ regulatory potential. These observations are especially relevant given the complex interplay between adipose tissue and the liver in obesity-driven metabolic dysfunction, underscoring the importance of a multi-tissue therapeutic strategy. By leveraging the distinct properties of natural products, as demonstrated by Gnetin C, we can explore new prophylactic and therapeutic approaches for obesity and diabetes.

Therefore, in this study, we administered oral Gnetin C to HFD-fed mice to determine whether this dimer functions as the principal active component of MSE, specifically through its regulation of both adipose tissue and the liver. Additionally, we aimed to elucidate its distinctive mode of action against obesity and diabetes. By uncovering the novel mechanisms of Gnetin C, we seek to highlight the broader potential of natural product-derived compounds in advancing both prophylactic and therapeutic strategies for metabolic disorders.

## Results

### Gnetin C improves body weight and fasting blood glucose level in HFD-fed mice

**Fig. 1:**
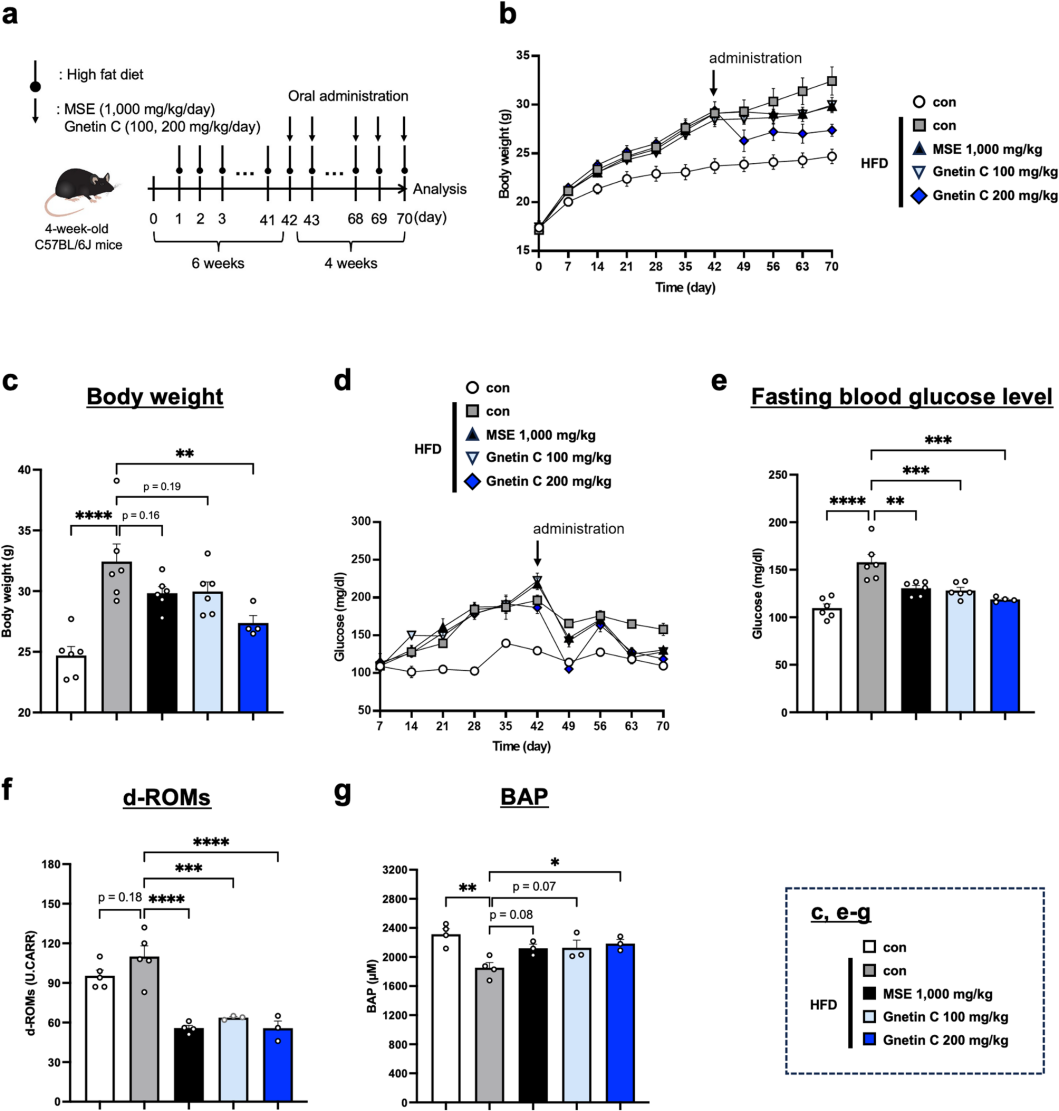
Gnetin C improves body weight and fasting blood glucose level in HFD-fed mice.** a** High-fat diet (HFD)-fed mice were orally administered with Gnetin C (100 or 200 mg/kg) or MSE (1,000 mg/kg) daily for 4 weeks. **b** Changes in body weight of HFD-fed mice treated with vehicle, Gnetin C (100 or 200 mg/kg), or MSE (1,000 mg/kg).　**c** Body weight measurements on day 70.　**d** Fasting blood glucose levels in HFD-fed mice treated with vehicle, Gnetin C (100 or 200 mg/kg), or MSE (1,000 mg/kg).　**e** Fasting blood glucose levels measured on day 70. **f**,** g** Serum d-ROMs (**f**) and BAP (**g**) levels of HFD-fed mice treated with vehicle, Gnetin C (200 mg/kg), or MSE (1,000 mg/kg) for 4 weeks, analyzed using the d-ROMs and BAP tests. Data are presented as mean ± SEM; 4–6 mice per group. Statistical significance was assessed using ANOVA followed by Dunnett’s test (*P < 0.05, **P < 0.01, ***P < 0.001, ****P < 0.0001).

Gnetin C, a resveratrol-like compound found in MSE and one of its key components, has previously been reported to exert beneficial effects on NAFLD^23^. Therefore, in this study, we investigated whether Gnetin C could improve the pathological conditions of an HFD-induced obesity and diabetes model, similar to MSE. To induce obesity and diabetes, 4-week-old C57BL/6J mice were fed a high-fat diet (HFD) *ad libitum* for 10 weeks. Starting from week 6 of HFD feeding, Gnetin C (100 or 200 mg/kg) and MSE (1,000 mg/kg) were administered daily by oral gavage for 4 weeks (Fig. 1a). Body weight and fasting blood glucose levels were measured over time to evaluate the effects of Gnetin C and MSE. Both the Gnetin C- and MSE-treated groups showed reductions in body weight (Fig. 1b, c). Notably, Gnetin C at 100 mg/kg exerted weight loss comparable to MSE at 1,000 mg/kg, while Gnetin C at 200 mg/kg led to even greater weight reduction. In contrast, administration of Gnetin C at 200 mg/kg in wild-type mice did not produce the same stable weight reduction seen in the model mice—that is, no weight loss was observed under normal conditions—suggesting that the weight loss induced by Gnetin C is attributable to its alleviation of metabolic toxicity under high‐fat diet challenge (Supplementary Fig. 1). Similarly, fasting blood glucose levels were normalized in both Gnetin C- and MSE-treated groups (Fig. 1d, e). To assess the impact of Gnetin C and MSE on systemic oxidative stress induced by obesity and diabetes, levels of oxidative stress marker d-ROMs and antioxidant capacity (BAP) were measured in serum. Both Gnetin C and MSE significantly reduced oxidative stress and enhanced antioxidant capacity (Fig. 1f, g). These findings indicate that Gnetin C exerts anti-obesity and anti-diabetic effects similar to those of MSE, suggesting that Gnetin C is the key active component of MSE.

### Gnetin C reduces fat accumulation and promotes APN multimerization in adipose tissue

**Fig. 2:**
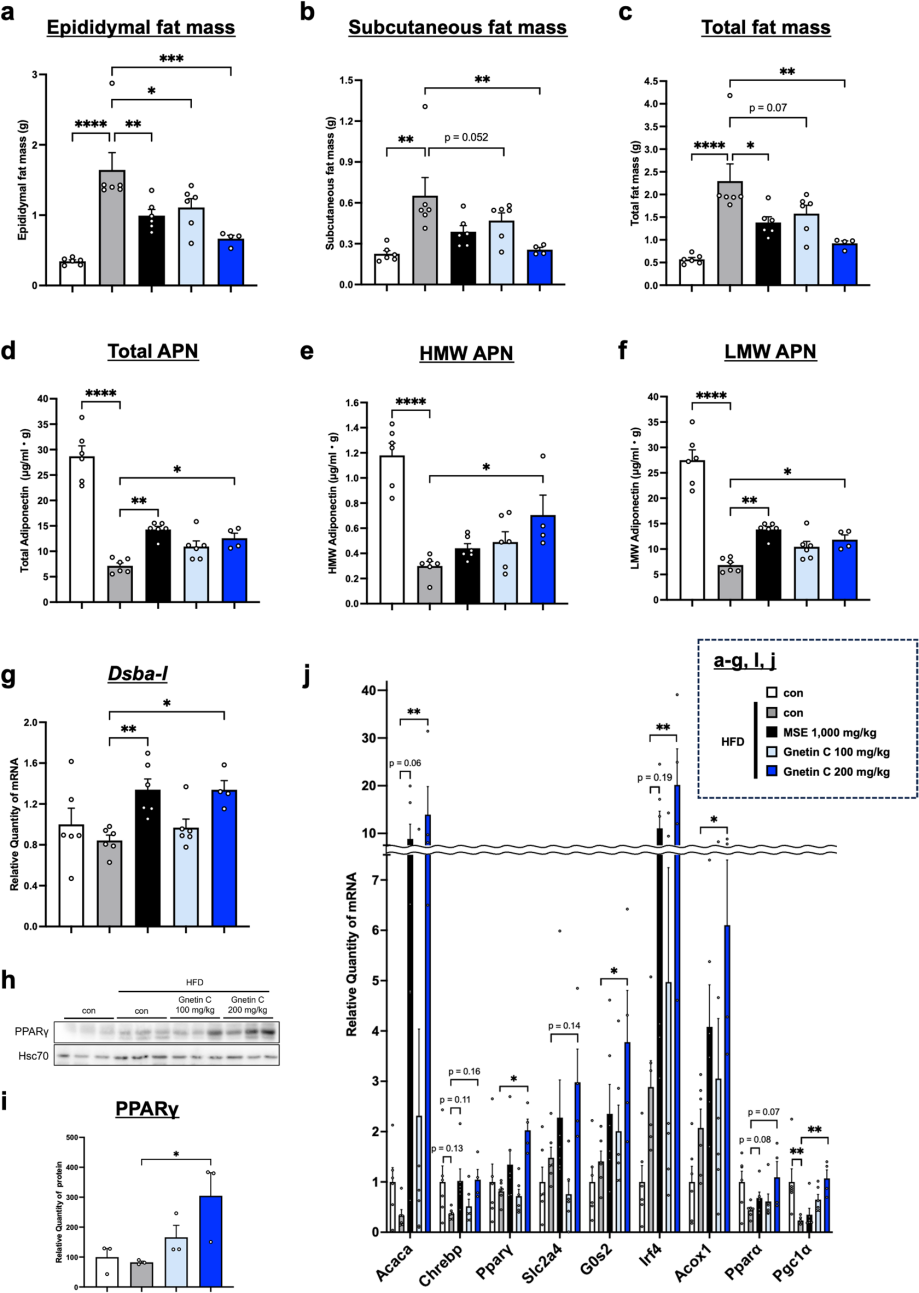
Gnetin C reduces fat accumulation and promotes APN multimerization in adipose tissue.** a-c** Epididymal fat mass (**a**), subcutaneous fat mass (**b**), and total fat mass (**c**) levels in HFD-fed mice treated with vehicle, MSE (1,000 mg/kg/day), or Gnetin C (100 or 200 mg/kg/day) for 4 weeks. **d-f** Serum total APN (**d**), HMW APN (**e**), and LMW APN (**f**) levels, adjusted for fat mass, in HFD-fed mice treated with vehicle, MSE (1,000 mg/kg), or Gnetin C (100 or 200 mg/kg). **g** Relative mRNA expression levels of Dsba-l in adipose tissue of HFD-fed mice treated with vehicle, MSE (1,000 mg/kg), or Gnetin C (100 or 200 mg/kg). **h**,** i** Relative protein levels of PPARγ in adipose tissue of HFD-fed mice treated with vehicle, MSE (1,000 mg/kg), or Gnetin C (100 or 200 mg/kg). **j** Relative mRNA levels of genes involved in fatty acid synthesis (Acaca, Chrebp), adipose differentiation (Pparγ, Slc2a4), lipolysis (G0s2, Irf4), and fatty acid β-oxidation (Acox1, Pparα, Pgc1α) in adipose tissue from HFD-fed mice treated with vehicle, MSE (1,000 mg/kg), or Gnetin C (100, 200 mg/kg). Data are presented as mean ± SEM; 4–6 mice per group. Statistical significance was determined using ANOVA followed by Dunnett’s test (*P < 0.05, **P < 0.01, ***P < 0.001, ****P < 0.0001).

To evaluate the effects of Gnetin C and MSE on fat accumulation, subcutaneous and epididymal fat were excised at the time of dissection, and their weights were measured. Both Gnetin C- and MSE-treated groups exhibited reduced subcutaneous and epididymal fat mass (Fig. 2a-c). Although Gnetin C conferred metabolic improvements similar to MSE, it remains unclear whether their mechanisms of action are identical. To assess the effects of Gnetin C and MSE on circulating APN and high-molecular-weight (HMW) APN levels, ELISA was performed using mouse serum. Because APN is secreted from adipocytes, the ELISA data were normalized to fat mass. Both Gnetin C and MSE increased total APN levels, and notably, Gnetin C significantly elevated HMW APN levels (Fig. 2d, e); moreover, the extent of this increase was more pronounced than that of LMW APN (total APN minus HMW APN) (Fig. 2f), suggesting that APN multimerization plays a crucial role in the metabolic improvements conferred by Gnetin C.

Further analysis revealed that Gnetin C did not alter the expression of endoplasmic reticulum protein 44 (Erp44), another factor involved in APN multimerization^24^, in adipose tissue. However, Gnetin C significantly increased the expression of DsbA-L and endoplasmic reticulum oxidoreductase 1α (Ero-1α)^25^, both key factors that promote APN multimerization (Fig. 2g, Supplementary Fig. 2a, b). Additionally, Gnetin C enhanced the expression of peroxisome proliferator-activated receptor γ (PPARγ)^26^, a known upstream regulator of DsbA-L and Ero-1α (Fig. 2h-j, Supplementary Fig. 3a, b). These findings suggest that Gnetin C promotes APN multimerization and exerts metabolic benefits by activating the PPARγ-DsbA-L-Ero-1α axis. Beyond these multimerization-related factors, we also assessed a panel of genes related to lipid metabolism. In adipose tissue, Gnetin C increased the expression of genes associated with fatty acid synthesis, adipocyte differentiation, lipolysis, and fatty acid β-oxidation (Fig. 2j).

### Gnetin C increases FGF21 expression by activating the upstream regulator Sirt1

**Fig. 3:**
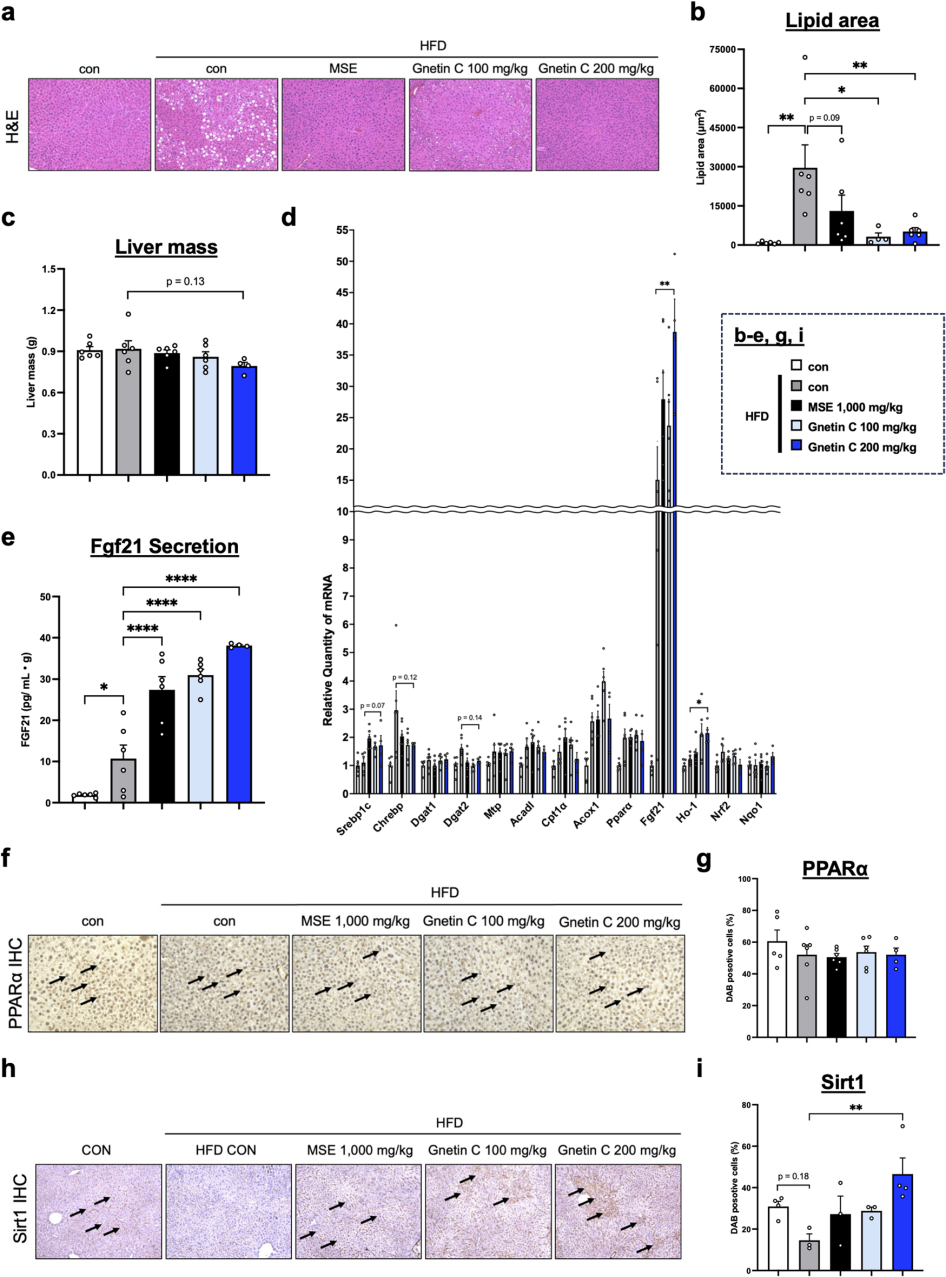
Gnetin C increases FGF21 expression by activating the upstream regulator Sirt1.** a**,** b** Measurement of lipid area in liver tissue using H&E staining in HFD-fed mice treated with vehicle, MSE (1,000 mg/kg/day), or Gnetin C (100 or 200 mg/kg/day) for 4 weeks. **c** Liver mass level in HFD-fed mice treated with vehicle, MSE (1,000 mg/kg/day), or Gnetin C (100 or 200 mg/kg/day) for 4 weeks. **d** Relative mRNA expression levels of genes related to fatty acid synthesis (Srebp1c, Chrebp), triglyceride synthesis (Dgat1, Dgat2, Mtp), fatty acid β-oxidation (Acadl, Cpt1α, Acox1, Pparα, Fgf21), and oxidative stress (Ho-1, Nrf2, Nqo1) in liver tissue of HFD-fed mice treated with vehicle, MSE (1,000 mg/kg), or Gnetin C (100 or 200 mg/kg). **e** Serum FGF21 levels, adjusted for liver mass, in HFD-fed mice treated with vehicle, MSE (1,000 mg/kg), or Gnetin C (100 or 200 mg/kg). **f**,** g** Representative images of IHC staining with an anti-PPARα antibody in liver tissue of HFD-fed mice treated with vehicle, MSE (1,000 mg/kg), or Gnetin C (100 or 200 mg/kg). **h**,** i** Representative images of IHC staining with an anti-Sirt1 antibody in liver tissue of HFD-fed mice treated with vehicle, MSE (1,000 mg/kg), or Gnetin C (100 or 200 mg/kg). Data are presented as mean ± SEM; 4–6 mice per group. Statistical significance was assessed using ANOVA followed by Dunnett’s test (*P < 0.05, **P < 0.01, ***P < 0.001, ****P < 0.0001).

To evaluate the effects of Gnetin C on hepatic lipid accumulation, H&E staining was performed. The results showed that Gnetin C reduced lipid accumulation in the liver (Fig. 3a, b). In addition, liver weight was not significantly affected by Gnetin C treatment (Fig. 3c). Next, to identify the key regulatory factors underlying the lipid-lowering effects of Gnetin C in the liver, we conducted a comprehensive analysis of genes involved in fatty acid synthesis, triglyceride synthesis, fatty acid β-oxidation, and oxidative stress. Among these genes, fibroblast growth factor 21 (Fgf21), which is associated with fatty acid β-oxidation, was significantly upregulated (Fig. 3d). FGF21, a hepatocyte secreted by the liver, is known for its potent metabolic regulatory effects on adipose tissue and other organs, making it a subject of intense research^27,28^. Measurement of circulating FGF21 levels by ELISA, normalized to liver weight, revealed that Gnetin C further enhanced the HFD-induced increase in FGF21 secretion (Fig. 3e). Considering that obesity is associated with increased FGF21 secretion and FGF21 resistance^29^, the additional upregulation of endogenous FGF21 may be crucial for the metabolic improvements observed in Gnetin C treatment.

To investigate the mechanism by which Gnetin C increases FGF21 expression, we focused on peroxisome proliferator-activated receptor α (PPARα)^27,^ a nuclear receptor known to regulate FGF21 expression. However, immunohistochemical analysis of PPARα in liver tissue showed that Gnetin C did not alter PPARα expression (Fig. 3f, g). We then turned our attention to sirtuin 1 (Sirt1), a NAD-dependent protein deacetylase known to induce FGF21 expression and improve fatty liver^30^. Previous studies have shown that resveratrol, a Sirt1 activator, enhances AMP-activated protein kinase (AMPK) activation and mitochondrial function in a Sirt1-dependent manner^31^. Given that Gnetin C is a resveratrol dimer, we hypothesized that Gnetin C might similarly influence Sirt1 expression. Immunohistochemical analysis of Sirt1 in liver tissue revealed that Gnetin C increased Sirt1 expression (Fig. 3h, i), suggesting that Sirt1 upregulation may contribute to the increase in FGF21 expression observed with Gnetin C treatment.

### Gnetin C suppresses fat accumulation in adipose and hepatic cells in vitro

**Fig. 4:**
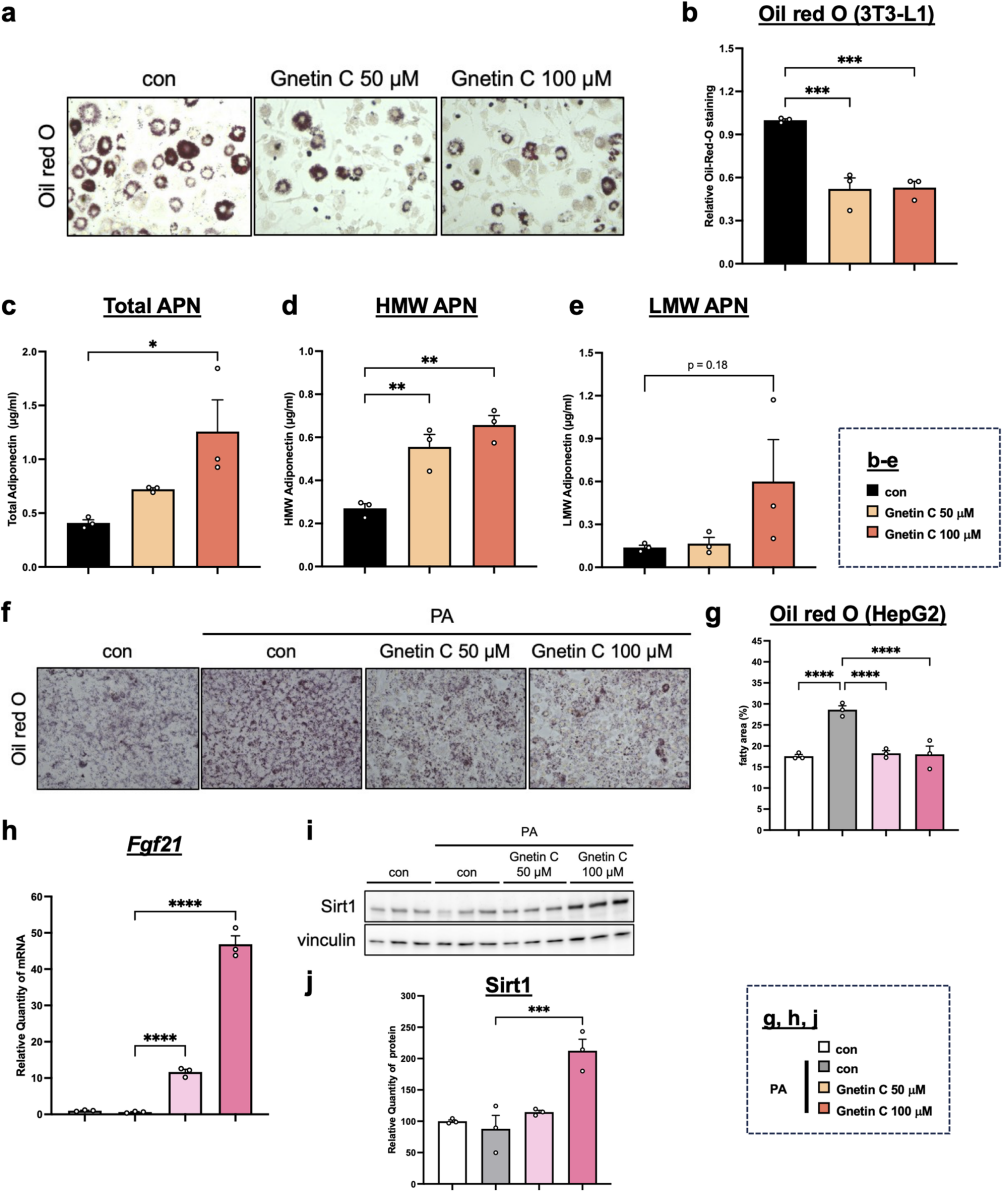
Gnetin C suppresses fat accumulation in adipose and hepatic cells in vitro. **a**,** b** Measurement of lipid area using Oil Red O staining in 3T3-L1 cells treated with 0.1% DMSO or Gnetin C (50 µM, 100 µM) for 6 h. **c-e** Serum total APN (**c**), HMW APN (**d**), and LMW APN (**e**) levels in 3T3-L1 cells treated with 0.1% DMSO or Gnetin C (50 µM, 100 µM) for 6 h. **f**,** g** Measurement of lipid area using Oil Red O staining in HepG2 cells treated with palmitic acid for 18 h, followed by 0.1% DMSO or Gnetin C (50 µM, 100 µM) for 6 h. **h** Relative mRNA expression levels of Fgf21 in HepG2 cells treated with palmitic acid for 18 h, followed by 0.1% DMSO or Gnetin C (50 µM, 100 µM) for 6 h. **i**,** j** Relative protein levels of Sirt1 in HepG2 cells treated with palmitic acid for 18 h, followed by 0.1% DMSO or Gnetin C (50 µM, 100 µM) for 6 h. Data are presented as mean ± SEM; 3 cell groups per condition. Statistical significance was assessed using ANOVA followed by Dunnett’s test (*P < 0.05, **P < 0.01, ***P < 0.001, ****P < 0.0001).

It remains unclear whether the effects of Gnetin C on adipose and liver tissues are direct or mediated indirectly through APN or FGF21^32–34^. To clarify this, we investigated the direct effects of Gnetin C in vitro. First, we used differentiated adipocytes derived from mouse 3T3-L1 preadipocyte. After 10 days of differentiation, 3T3-L1 adipocytes were treated with Gnetin C. Gnetin C not only reduced lipid accumulation (Fig. 4a, b), but it also produced a profile similar to the in vivo results: a slight increase in LMW APN levels in the culture supernatant, but a marked increase in HMW APN (Fig. 4c-e). These findings suggest that Gnetin C directly promotes APN multimerization in adipose tissue, thereby suppressing lipid accumulation.

Next, we used HepG2 cells, a human hepatoma-derived cell line treated with the saturated fatty acid palmitic acid to induce lipid accumulation. Similar to 3T3-L1 adipocytes, Gnetin C treatment reduced lipid accumulation in HepG2 cells (Fig. 4f and g). Moreover, the expression of Fgf21 and Sirt1 was upregulated (Fig. 4h-j, Supplementary Fig. 4a, b), suggesting that Gnetin C directly activates the Sirt1-FGF21 axis in liver tissue to suppress lipid accumulation.

### Gnetin C enhances hepatic SIRT1 activity and directly activates SIRT1 in vitro

**Fig. 5:**
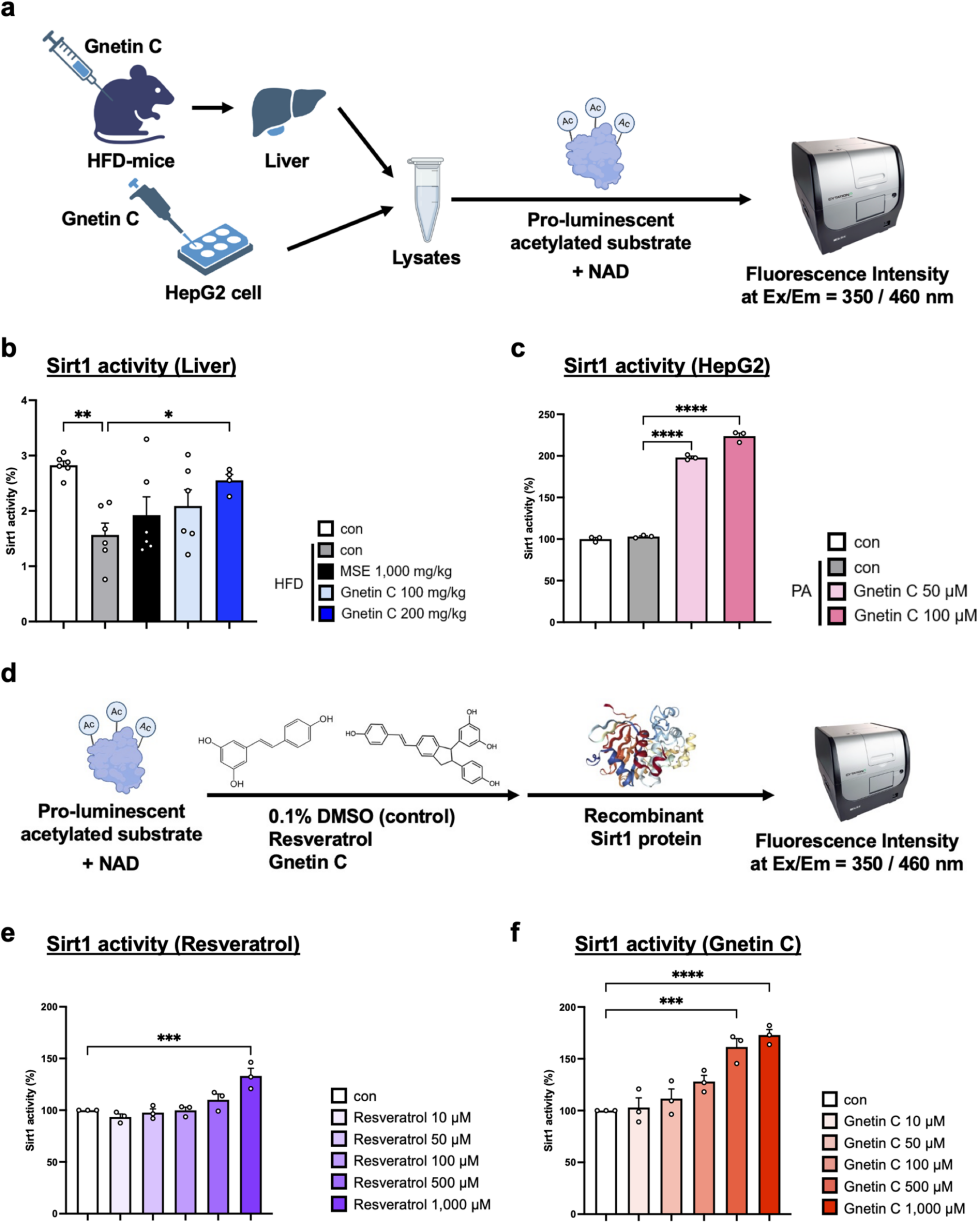
Gnetin C enhances hepatic SIRT1 activity and directly activates SIRT1 in vitro. **a** Schematic diagram of the Sirt1 activity assay. When lysates are combined with a pro-luminescent acetylated substrate and NAD, the Sirt1 enzyme in the lysates deacetylates the substrate in an NAD-dependent manner, resulting in fluorescence emission. **b** Sirt1 deacetylase activity was measured using a Sirt1 activity assay kit in liver lysates from HFD-fed mice treated with vehicle, MSE (1,000 mg/kg), or Gnetin C (100 or 200 mg/kg). **c** Sirt1 deacetylase activity was measured using a Sirt1 activity assay kit in HepG2 cells treated with palmitic acid for 18 h, followed by 0.1% DMSO or Gnetin C (50 µM, 100 µM) for 6 h. **d** Schematic diagram of the Sirt1 activator screening assay. **e** Sirt1 deacetylase activity was measured using a Sirt1 activity assay kit in the presence of 0.1% DMSO or resveratrol (10, 50, 100, 500, 1,000 µM). **f** Sirt1 deacetylase activity was measured using a Sirt1 activity assay kit in the presence of 0.1% DMSO or Gnetin C (10, 50, 100, 500, 1,000 µM). Data are presented as mean ± SEM; 3 cell groups per condition for cell-based assays and 4–6 mice per group for animal studies. Statistical significance was assessed using ANOVA followed by Dunnett’s test (*P < 0.05, **P < 0.01, ***P < 0.001, ****P < 0.0001).

Previous studies have reported that resveratrol enhances Sirt1 activity by directly binding to it^34^. To investigate whether Gnetin C enhances Sirt1 activity through similar mechanisms, a Sirt1 activity assay was performed. This assay measures fluorescence emitted by acetylated substrates upon their deacetylation by Sirt1 in the presence of NAD⁺ and tissue or cell lysates (Fig. 5a). When liver lysates from Fig. 3 were tested, Sirt1 activity was found to be increased in the Gnetin C-treated group (Fig. 5b). Similarly, Sirt1 activity was elevated in lysates from HepG2 cells treated with Gnetin C (Fig. 5c), consistent with the increase in Sirt1 expression (Fig. 4i, j). These findings indicate that Gnetin C enhances Sirt1 activity in vivo, particularly in liver tissue. Next, to determine whether Gnetin C enhances Sirt1 activity through a direct binding mechanism, a Sirt1 activator screening assay was performed. This assay evaluates direct activation by incubating acetylated substrates and NAD⁺ with candidate direct activators in the presence of recombinant Sirt1 protein (Fig. 5d). As a positive control, the extent of Sirt1 activation by resveratrol was compared with a negative control (0.1% DMSO). Consistent with previous reports, resveratrol exhibited a concentration-dependent direct activation of Sirt1 (Fig. 5e). When tested under the same condition, Gnetin C also demonstrated a concentration-dependent direct activation of Sirt1, similar to resveratrol. Notably, under these conditions, Gnetin C induced a higher level of Sirt1 activation than resveratrol (Fig. 5f), indicating that Gnetin C can directly activate Sirt1 in vitro and may exhibit stronger activity than resveratrol in this context.

### Gnetin C enhances expression of FGF21 receptors in hepatocytes and adipocytes: implications for autocrine and paracrine effects

**Fig. 6:**
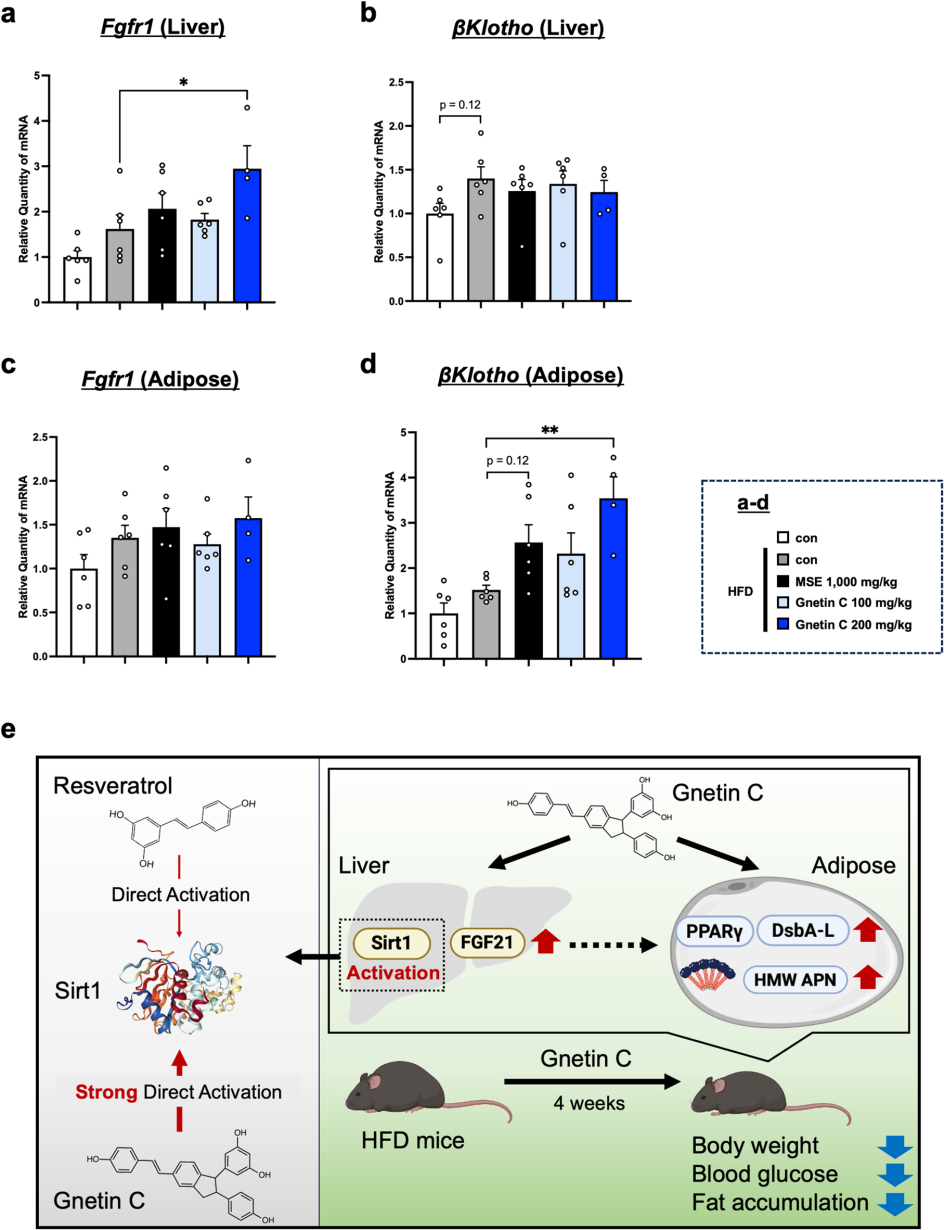
Gnetin C enhances expression of FGF21 receptors in hepatocytes and adipocytes: Implications for autocrine and paracrine effects.** a**,** b** Relative mRNA expression levels of Fgfr1 (**a**) and βKlotho (**b**) in liver tissue of HFD-fed mice treated with vehicle, MSE (1,000 mg/kg), or Gnetin C (100, 200 mg/kg). **c**,** d** Relative mRNA expression levels of Fgfr1 (**c**) and βKlotho (**d**) in adipose tissue of HFD-fed mice treated with vehicle, MSE (1,000 mg/kg), or Gnetin C (100, 200 mg/kg). **e** Schematic overview of the study. Data are presented as mean ± SEM; 4–6 mice per group. Statistical significance was assessed using ANOVA followed by Dunnett’s test (*P < 0.05, **P < 0.01).

Previous studies have reported that FGF21 suppresses hepatic lipid accumulation through autocrine action^35^, and it exerts paracrine effects on various organs. Among these paracrine effects, its impact on adipose tissue is particularly significant, as FGF21 has been shown to increase APN levels in adipose tissue, thereby improving glucose and lipid metabolism^36,37^. Additionally, FGF21 has been reported to activate the PPARγ-DsbA-L-Ero-1α pathway^36^ and increase HMW APN levels^38^. These findings suggest that Gnetin C may exert its effects not only through direct action on adipose tissue but also by enhancing the paracrine effects of FGF21, promoting APN multimerization in adipose tissue. To investigate the potential role of FGF21-mediated autocrine and paracrine signaling in the metabolic improvements induced by Gnetin C, we analyzed the expression of FGF21 receptors in adipose and liver tissues. FGF21 receptors consist of a cell surface receptor complex comprising fibroblast growth factor receptor 1 (FGFR1) and the co-receptor protein βKlotho^39^, and the expression levels of both components were examined. In liver tissue, Gnetin C increased FGFR1 expression but did not affect βKlotho expression (Fig. 6a, b). These findings, together with the results in Fig. 5, suggest that FGF21 upregulated by Gnetin C contributes to the suppression of hepatic lipid accumulation primarily through autocrine action. In adipose tissue, Gnetin C increased βKlotho expression but did not alter FGFR1 expression (Fig. 6c, d). This observation suggests that, in addition to Gnetin C’s dual adipose and hepatic actions, FGF21 upregulated by Gnetin C may act on adipose tissue *via* paracrine signaling, activating the PPARγ-DsbA-L-Ero-1α axis and thereby partially contributing to APN multimerization (Fig. 6e).

## Discussion

In our previous work, we found that MSE alleviates obesity-related metabolic disorders by promoting APN multimerization^19^. To identify the active component responsible for this effect, we focused on Gnetin C, a principal compound found in MSE. Here, we demonstrate that Gnetin C exerts anti-obesity and anti-diabetic effects similar to MSE, primarily by enhancing APN multimerization through DsbA-L upregulation in adipose tissue, consistent with the action of MSE. Moreover, our results showed that Gnetin C also increases the expression of Ero-1α, another critical factor for APN multimerization. Like DsbA-L, Ero-1α catalyzes disulfide bond formation in APN, facilitating its secretion from the endoplasmic reticulum (ER)^40^. Conversely, Erp44, whose expression remained unchanged, plays an essential role in the quality control of APN by binding to APN to form LMW APN and then dissociating upon disulfide bond formation with Ero-1α, thereby enabling the formation of HMW APN^24^. These findings suggest that Gnetin C predominantly influences HMW APN formation by regulating APN’s high-order multimerization factors rather than contributing to LMW APN formation. In addition, we investigated PPARγ, an upstream regulator of these APN multimerization factors^26^. Although PPARγ is known to regulate the expression of DsbA-L, Ero-1α, and Erp44, our results imply that additional upstream regulators may be involved in the Gnetin C-mediated upregulation of DsbA-L and Ero-1α, necessitating further study.

In addition to multimerization-related factors, the gene expression analysis revealed that Gnetin C increased the expression of several genes involved in lipid metabolism in adipose tissue. Some of these, such as Slc2a4 (GLUT4) and G0s2, are recognized downstream targets of PPARγ^41,42^, consistent with the observed activation of the PPARγ-DsbA-L-Ero-1α axis. The induction of Acaca and Chrebp, both lipogenic genes, likely reflects activation of *de novo* lipogenesis programs during adipocyte differentiation. Given that GLUT4 is a known PPARγ target and can regulate Chrebp activity^43^, and that Chrebp directly controls Acaca expression, these changes may represent downstream consequences of PPARγ activation that also contribute to systemic insulin sensitivity. In parallel, Irf4 and its downstream coactivator Pgc1α, together with Pparα and its target gene Acox1, were also upregulated, pointing to enhanced lipolysis, mitochondrial biogenesis, and fatty acid oxidation^44,45^. These findings suggest that Gnetin C may influence adipose metabolism not only through PPARγ-dependent pathways but also *via* additional transcriptional programs.

We also demonstrated that Gnetin C ameliorates obesity and diabetes partly by increasing FGF21 expression and secretion in the liver. FGF21 functions as a hepatocyte with both autocrine and paracrine effects. However, to clarify the contributions of these mechanisms, further work using tissue-specific FGF21 receptor-deficient models is required. While our study focused on adipose tissue, the most reported target of FGF21’s paracrine effects, FGF21 also promotes β-cell proliferation and function in the pancreas^46^, improves insulin sensitivity in muscle^47^, and activates sympathetic nerves through the hypothalamus^48^, suggesting additional organ-level contributions to the improvements in obesity and diabetes mediated by Gnetin C. Furthermore, although we confirmed the upregulation and activation of Sirt1, an upstream regulator of FGF21, other factors such as PPARα, activating transcription factor 4 (ATF4)^49^, and peroxisome proliferator-activated receptor gamma coactivator-1 α (PGC-1α)^50^ may also play roles and warrant further investigation.

Although the precise molecular mechanisms underlying the SIRT1-mediated regulation of FGF21 were not directly addressed in this study, previous reports provide important insights. For example, SIRT1 has been shown to regulate FGF21 expression in the liver^51^, and more recently, the metabolic benefits of resveratrol have been reported to depend on FGF21 signaling^52^. These findings indicate that the SIRT1-FGF21 regulatory axis is increasingly recognized as a key pathway in metabolic regulation. Our present data support this concept by demonstrating Gnetin C-induced activation of SIRT1 accompanied by FGF21 upregulation. Nevertheless, the mechanistic basis of this regulation remains unclear. Future studies incorporating chromatin immunoprecipitation (ChIP) assays and analyses of histone acetylation at the FGF21 promoter will be crucial for determining whether SIRT1 directly regulates FGF21 transcription *via* chromatin remodeling. Moreover, the direct causal relationships among Gnetin C, SIRT1 activation, FGF21 induction, and APN multimerization remain to be established and should be confirmed in additional mechanistic studies.

Our previous work has shown that Gnetin C improves blood lipid profiles, including significant reductions in plasma triglyceride and total cholesterol levels, and attenuates hepatic lipid accumulation in an NAFLD mouse model. In addition, that study showed suppression of hepatic genes related to triglyceride synthesis and transport (e.g., Dgat1, Dgat2, Mtp)^23^. Importantly, our data (Fig. 3d) likewise demonstrate a significant downregulation of Dgat2 expression in the liver, which is consistent with the reported mechanism of Gnetin C. Together, these convergent findings strengthen the notion that Gnetin C contributes to the lipid-lowering effects of MSE through suppression of triglyceride synthesis and improvement of systemic lipid metabolism.

Despite ongoing debate regarding the direct activation of Sirt1 by resveratrol, our Sirt1 activator screening assay confirmed its concentration-dependent activation^33,34^, in agreement with previous studies^53^. Notably, we demonstrated for the first time that Gnetin C, a resveratrol dimer, exhibits stronger activity than resveratrol in our in vitro assay, supporting its potential as a SIRT1 activator. Importantly, the comparison between resveratrol and Gnetin C in this study was made using a recombinant SIRT1-based screening assay (Fig. 5d-f), which directly assesses the intrinsic ability of compounds to activate SIRT1 and is not influenced by pharmacokinetic properties. Thus, the stronger activation observed for Gnetin C reflects a direct molecular effect. Furthermore, prior reports have shown greater in vivo retention of Gnetin C and superior improvements in body weight, liver weight, and insulin sensitivity relative to resveratrol in a NAFLD model^23,52^. These findings complement our in vitro data and collectively support Gnetin C as a promising activator of SIRT1. Additional assessments beyond the screening assay remain necessary to fully validate these findings.

Our results suggest that Gnetin C may promote APN multimerization not only through activation of the PPARγ-DsbA-L-Ero-1α axis in adipose tissue but also indirectly *via* the Sirt1-FGF21 axis in the liver, highlighting its promise as a novel agent for metabolic disease therapy. Although Gnetin C shows promise for improving NAFLD^23^ and potentially mitigating periodontal disease through antioxidant effects^16^, preventing Alzheimer’s disease^54^, and exerting anti-inflammatory actions^55^, these findings remain limited and require further investigation. Gnetin C also exhibits potent antioxidant properties, contributing to pronounced anti-cancer effects^14,15,56^, underscoring its broader clinical relevance. However, our data reveal that high-dose Gnetin C administration can induce gastrointestinal symptoms in mice, such as diarrhea and lymphangiectasia-like conditions (described in the Methods section). Given that Gnetin C is known to have strong bioactivity (including anticancer properties), there are legitimate safety concerns at elevated concentrations, necessitating cautious evaluation. The small sample size of the high-dose group constitutes a limitation of the present study, and further investigations are warranted to validate the safety profile of Gnetin C.

In contrast, MSE demonstrates stable efficacy with fewer gastrointestinal side effects. Consistent with previous safety assessments, our current results confirm that MSE exhibits a robust safety profile^11,18^, distinctly different from the adverse effects observed with high-dose Gnetin C administration. Intriguingly, our recent study showed that MSE administration leads to high circulating levels of Gnetin C and its glycosylated derivatives, including Gnemonoside A. This finding prompts further exploration into whether non-Gnetin C components, or particularly Gnetin C glycosides, contribute to MSE’s overall therapeutic effects^12^. In any case, both MSE and Gnetin C exert beneficial actions through direct impact on the liver and adipose tissue and through inter-organ mechanisms involving the liver-adipose axis. These insights offer valuable information for developing multi-target treatment strategies against metabolic disorders.

## Methods

### Materials and chemicals

MSE powder (YMP-M-181201), containing 0.07% RES, 2.41% gnetin C, 23.9% gnemonoside A, 4.51% gnemonoside D, and 9% dextrin as an excipient, was obtained from the Yamada Bee Company, Inc. (Okayama, Japan). Gnetin C (purity: >98%, HPLC) was prepared according to a previously described method^11^.

### Experimental animals and treatments

C57BL/6J mice were housed in the animal experimentation facility at Kumamoto University in accordance with the guidelines of the university’s Animal Facility Center. The mice were maintained under a 12-hour light/dark cycle at a room temperature of 20 °C. For HFD, we purchased the chow from Oriental Yeast Co., Ltd. (Tokyo, Japan), and replaced once weekly with 300 g of fresh chow after an overnight fasting period. The composition of the HFD was 25% casein, 20% safflower oil, 14% beef tallow, 8% dextrin, 7% lactose, 7% sucrose, and 5% cellulose. Five-week-old mice were randomly divided into five groups and fed with the HFD for six weeks. After ensuring that the average body weight of each group was similar at the end of the sixth week, the mice were fed HFD for an additional four weeks. MSE was administered once daily for four weeks at a dose of 1,000 mg/kg by oral gavage using a gastric tube. Similarly, Gnetin C was administered once daily for four weeks at doses of 100 mg/kg or 200 mg/kg by oral gavage using a gastric tube. The Gnetin C dose was determined based on the amount of Gnetin C and its glycosides present in 1,000 mg/kg of MSE. Notably, 1,000 mg/kg of MSE contains not only Gnetin C but also its glycosides (Gnemonosides), and assuming complete conversion of these glycosides to Gnetin C, while also accounting for inter-lot variations, the effective dose is estimated to fall within the range of 100 to 200 mg/kg. Therefore, two dose levels of 100 and 200 mg/kg were established. In the high-concentration group (200 mg/kg), gastrointestinal symptoms such as diarrhea and lymphangiectasia-like conditions were frequently observed in mice. Before administration, the test compounds were thoroughly suspended using a mortar and pestle.　Blood glucose levels were measured using an Accu-Chek Compact glucometer (Roche, Basel, Switzerland), with blood samples collected *via* tail vein puncture. Fasting blood glucose levels were measured after more than 12 h of fasting, during which water was freely available. Anesthesia was induced in mice using an intraperitoneal injection of three types of mixed anesthesia (0.75 mg/kg of medetomidine [NIPPON ZENYAKU KOGYO CO., LTD., Fukushima, Japan], 4 mg/kg of midazolam [Sandoz K. K., Tokyo, Japan] and 5 mg/kg of butorphanol [Meiji Animal Health Co., Ltd., Kumamoto, Japan]). Following confirmation of a surgical plane of anesthesia, euthanasia was performed by exsanguination via cardiac puncture. These procedures adhered to the 2020 AVMA Guidelines for the Euthanasia of Animals. The animal study was conducted in accordance with the ARRIVE guidelines (Animal Research: Reporting of In Vivo Experiments). All animal procedures were approved by the Animal Welfare Committee of Kumamoto University (#A2024-050, to T.S.) and conducted in compliance with the relevant national and institutional guidelines for the care and use of laboratory animals.

### Cell culture

3T3-L1 cells (RIKEN BRC, Japan) were used to evaluate the direct effects of Gnetin C on adipocytes. The cells were cultured in DMEM (High Glucose) medium (Wako, Osaka, Japan) supplemented with 10% Fetal Calf Serum (FCS) and 1% antibiotics (P/S; Penicillin G [100 U/mL] and Streptomycin [100 µg/mL]). To induce differentiation, the cells were maintained at confluence for two days, then the medium was replaced with 3T3-L1 adipocyte differentiation medium (KAC, Kyoto, Japan) and cultured for three days. Subsequently, the cells were cultured in a 3T3-L1 adipocyte culture medium (KAC, Kyoto, Japan) for seven days. HepG2 cells were obtained from the American Type Culture Collection (ATCC) and used to evaluate the direct effects of Gnetin C on hepatocytes. The cells were cultured in DMEM (Low Glucose) medium (Wako, Osaka, Japan) supplemented with 10% Fetal Bovine Serum (FBS) and 1% antibiotics (P/S; Penicillin G [100 U/mL] and Streptomycin [100 µg/mL]). To induce lipid accumulation in HepG2 cells, the cells were treated with 0.3 mM palmitic acid (Thermo Fisher, Massachusetts, USA) for 18 h. For maintenance culture, the culture dishes (3003 dishes, Falcon, Texas, USA) were pre-coated with Cell Matrix type I-C (Nitta Gelatin, Osaka, Japan) and incubated at 37 °C for at least 30 min before use. All cells were cultured in a humidified incubator at 37 °C with 5% CO₂.

### RNA isolation and quantitative RT-qPCR

Total RNA was extracted from mouse adipose and liver tissues, 3T3-L1 cells, and HepG2 cells using RNAiso Plus^®^. For reverse transcription (RT) reactions, the PrimeScript^®^ RT Master Mix (Perfect Real Time) kit (Takara Bio, Shiga, Japan) was used according to the manufacturer’s protocol. Subsequently, PCR reactions were performed using the resulting cDNA, TB Green Premix Ex Taq II (Tli RNaseH Plus) (Takara Bio, Shiga, Japan), and gene-specific primers under the following conditions: 95 °C for 3 min; followed by 40 cycles of [95 °C for 10 s, 65 °C for 1 min]; then a plate read. The reactions were carried out using a CFX Connect™ Real-Time PCR Detection System (Bio-Rad Laboratories, California, USA). To normalize mRNA expression levels across samples and wells, Gapdh was used as the internal control for mouse tissues, while 18 S rRNA was used for cells. The primer sequences for each target gene in mice are shown in Table 1.

**Table 1:** Sequences of primers for quantitative RT-PCR.

Primer	Sequence
Mouse Dsba-l forward	5’-GGTCCTATGCAGATACCAACAC-3’
Mouse Dsba-l reverse	5’-GTACTGGCCTTTTCGGGGAA-3’
Mouse Ero-1α forward	5’-GGACTGTGTTGGCTGCTTCAAG-3’
Mouse Ero-1α reverse	5’-GCTGGAACTCATAACTTGGTCCG-3’
Mouse Erp44 forward	5’-GATGACTGTGCCTTCCTTTCTGC-3’
Mouse Erp44 reverse	5’-CAAGTACACCATGTCTGGCGCA-3’
Mouse Srebp1c forward	5’-CCCTTGACTTCCTTGCTGCA-3’
Mouse Srebp1c reverse	5’-GCGTGAGTGTGGGCGAATC-3’
Mouse Chrebp forward	5’-GAGTGCTTGAGCCTGGCTTACA-3’
Mouse Chrebp reverse	5’-GCTCTCCAGATGGCGTTGTTCA-3’
Mouse Dgat1 forward	5’-GGTTCCGTGTTTGCTCTGGCAT-3’
Mouse Dgat1 reverse	5’-CCACTGACCTTCTTCCCTGTAG-3’
Mouse Dgat2 forward	5’-CTGTGCTCTACTTCACCTGGCT-3’
Mouse Dgat2 reverse	5’-CTGGATGGGAAAGTAGTCTCGG-3’
Mouse Mtp forward	5’-CCAGGAAAGGTTCCTCTATGCC-3’
Mouse Mtp reverse	5’-GACTCTCTGATGTCGTTGCTTGC-3’
Mouse Acadl forward	5’-GGCGATTTCTGCCTGTGAGTTC-3’
Mouse Acadl reverse	5’-GCTGTCCACAAAAGCTCTGGTG-3’
Mouse Cpt1α forward	5’-AGGACCCTGAGGCATCTATT-3’
Mouse Cpt1α reverse	5’-ATGACCTCCTGGCATTCTCC-3’
Mouse Pparγ forward	5’-GTAACTGTCGGTTTCAGAAGTGCC-3’
Mouse Pparγ reverse	5’-ATCTCCGCCAACAGCTTCTCCT-3’
Mouse Slc2a4 forward	5’-GGTGTGGTCAATACGGTCTTCAC-3’
Mouse Slc2a4 reverse	5’-AGCAGAGCCACGGTCATCAAGA-3’
Mouse G0s2 forward	5’-GCTAGTGAAGCTATACGTGCTGG-3’
Mouse G0s2 reverse	5’-GGACTGCTGTTCACACGCTTCC-3’
Mouse Irf4 forward	5’-GAACGAGGAGAAGAGCGTCTTC-3’
Mouse Irf4 reverse	5’-GTAGGAGGATCTGGCTTGTCGA-3’
Mouse Acox1 forward	5’-GTCTCCGTCATGAATCCCGA-3’
Mouse Acox1 reverse	5’-TGCGATGCCAAATTCCCTCA
Mouse Pparα forward	5’-TATTCGGCTGAAGCTGGTGTAC-3’
Mouse Pparα reverse	5’-CTGGCATTTGTTCCGGTTCT-3’
Mouse Pgc1α forward	5’-TATGGAGTGACATAGAGTGTGCT-3’
Mouse Pgc1α reverse	5’-CCACTTCAATCCACCCAGAAAG–3’
Mouse Fgf21 forward	5’-ATCAGGGAGGATGGAACAGTGG-3’
Mouse Fgf21 reverse	5’-AGCTCCATCTGGCTGTTGGCAA-3’
Human Fgf21 forward	5’-CTGCAGCTGAAAGCCTTGAAGC-3’
Human Fgf21 reverse	5’-GTATCCGTCCTCAAGAAGCAGC-3’
Mouse Ho-1 forward	5’- GCCACCAAGGAGGTACACAT-3’
Mouse Ho-1 reverse	5’- GCTTGTTGCGCTCTATCTCC − 3’
Mouse Nrf2 forward	5’- CACTCCAGCGAGCAGGCTAT − 3’
Mouse Nrf2 reverse	5’- CTGGGACTGTAGTCCTGGCG − 3’
Mouse Nqo-1 forward	5’- TTCTGTGGCTTCCAGGTCTT − 3’
Mouse Nqo-1 reverse	5’- AGGCTGCTTGGAGCAAAATA − 3’
Mouse Fgfr1 forward	5’-GCCTCACATTCAGTGGCTGAAG-3’
Mouse Fgfr1 reverse	5’-AGCACCTCCATTTCCTTGTCGG-3’
Mouse βKlotho forward	5’-GAAAGAGTCCACGCCAGACATG-3’
Mouse βKlotho reverse	5’-CAGGTGAGGATCGGTAAACTGC-3’
Mouse Gapdh forward	5’-CCTGGAGAAACCTGCCAAGTATG-3’
Mouse Gapdh reverse	5’-GGTCCTCAGTGTAGCCCAAGATG-3’
Mouse 18 s forward	5’- GTAACCCGTTGAACCCCATT − 3’
Mouse 18 s reverse	5’- CCATCCAATCGGTAGTAGCG − 3’
Human 18 s forward	5’-CGGCTACCACATCCAAGGAA − 3’
Human 18 s reverse	5’-GCTGGAATTACCGCGGCT − 3’

### Protein isolation and western blotting

Proteins were extracted from mouse adipose tissue and HepG2 cells using RIPA buffer (50 mM Tris-HCl [pH 7.5], 150 mM NaCl, 1 mg/mL sodium deoxycholate, 1 mM Na₃VO₄, and 1% protease inhibitor cocktail). Protein concentrations were measured using the BCA method. Next, proteins were separated on 8% or 10% SDS-PAGE and transferred to a PVDF membrane (250 mA, 1.5 h). The membrane was then blocked for 1 h with 0.05% PBS-Tween containing 5% skim milk. After washing in 0.05% PBS-Tween, the membrane was incubated at room temperature for 1 h with primary antibodies diluted in Can Get Signal^®^ Solution 1 (TOYOBO, Osaka, Japan). Following the primary antibody incubation, the membrane was washed with 0.05% PBS-Tween and incubated at room temperature for 1 h with secondary antibodies diluted in Can Get Signal^®^ Solution 2 (TOYOBO, Osaka, Japan). After this incubation, the membrane was washed with 0.05% PBS-Tween, treated with Super Signal^®^ West Pico chemiluminescent substrate (Thermo Fisher, Massachusetts, USA), and signals were detected using the Vilber Bio Imaging Fusion system (Vilber Lourmat, Collégien, France). The detected bands were quantified using ImageJ software. Primary antibodies used were anti-PPARγ (sc-7273, Santa Cruz, Texas, USA) and anti-Sirt1 (sc-15404, Santa Cruz, Texas, USA). Anti-Hsc70 (ADI-SPA-815, Enzo Life Sciences, New York, USA) and anti-vinculin (sc-73614, Santa Cruz, Texas, USA) were used as loading controls.

### Enzyme-linked immunosorbent assay (ELISA)

Serum levels of total APN and HMW-APN were quantified using a Mouse HMW & Total APN ELISA kit (ALPCO, New Hampshire, USA) according to the manufacturer’s protocol. Similarly, FGF21 levels were measured using a Mouse/Rat FGF21 ELISA kit (R&D Systems, Minnesota, USA), following the manufacturer’s instructions. For these measurements, serum was obtained by centrifuging blood collected from the inferior vena cava of mice at 5,000 rpm and 4 °C for 30 min. Cell culture supernatants were also used for the assays.

### d-ROMs and BAP test

Plasma levels of diacron-Reactive Oxygen Metabolites (d-ROMs) and Biological Antioxidant Potential (BAP) were measured using the Free Carrio Duo system (WISMERLL, Tokyo, Japan) and the corresponding d-ROMs and BAP test kits (WISMERLL, Tokyo, Japan)^57^, according to the manufacturer’s protocol. For sample preparation, serum was obtained by centrifuging blood collected from the inferior vena cava of mice at 5,000 rpm and 4 °C for 30 min.

### Hematoxylin & Eosin (H&E) staining and quantification

Mouse livers were excised, washed with PBS (-), and immersed in 10% neutral buffered formalin at room temperature for 12 h. Following formalin fixation, the tissues were washed three times with PBS (-) and dehydrated with ethanol. Subsequent steps, including paraffin embedding and H&E staining, were outsourced to K.I. Steiner (Kumamoto, Japan). Stained images were captured using a BZ-X710 microscope (Keyence, Osaka, Japan), and lipid droplet quantification was performed using ImageJ software.

### Oil red O staining and quantification

After removing the culture medium from HepG2 and 3T3-L1 cells, the cells were fixed with 4% paraformaldehyde, then washed and dehydrated with 60% isopropanol. Subsequently, the cells were treated with a Oil red O staining solution and incubated in the dark for 20 min. After removing the staining solution, Milli-Q (MQ) water was added, and stained images were captured using a BZ-X710 microscope (Keyence, Osaka, Japan). ImageJ software was used for further analysis and quantification of the stained images.

### Immunohistochemical (IHC) staining

Paraffin-embedded liver tissue sections prepared for IHC staining were deparaffinized and immersed in Target Retrieval Solution (DAKO, Glostrup, Denmark). Antigen retrieval was performed using an autoclave at 121 °C for 20 min. To prevent non-specific staining, the VECTOR M.O.M. Immunodetection kit (VEC, California, USA) was used. The sections were incubated overnight at 4 °C in the dark with a 1:100 dilution of an anti-PPARα antibody (sc-398394, Santa Cruz, Texas, USA) and an anti-Sirt1 antibody (sc-15404, Santa Cruz, Texas, USA). On the following day, the sections were treated with M.O.M. Biotinylated Anti-Mouse IgG Reagent (VEC, California, USA), and the reaction was visualized using a diaminobenzidine (DAB) substrate system (Nichirei, Tokyo, Japan). Stained images were acquired using a Nano Zoomer S20 Digital Slide Scanner (Nikon, Tokyo, Japan), and the number of PPARα^+^ and Sirt1^+^ cells was quantified using HALO software (Indica Labs, Corrales, USA).

### Quantification of Sirt1 activity assay and Sirt1 activator screening assay

SIRT1 activity was assessed using a fluorogenic peptide substrate bearing an N-terminal fluorophore and a C-terminal quencher. Upon deacetylation by active SIRT1 in the presence of NAD⁺, the concurrently supplied protease (Developer) cleaves the substrate, separating the quencher from the fluorophore and generating fluorescence, thereby enabling quantification of deacetylase activity. For the SIRT1 activity assay, MQ water and kit components (SIRT1 Assay Buffer, Fluoro-Substrate Peptide, NAD⁺, and Developer) were added to microtiter plate wells, followed by the addition of liver tissue and HepG2 cell lysates, and the plate was incubated at room temperature. Fluorescence was measured 60 min after lysate addition at Ex/Em = 350/460 nm using a BioTek Cytation 5 plate reader (Agilent Technologies, CA, USA). This protocol quantifies NAD⁺-dependent deacetylase activity present in lysates and thus reports SIRT1 activity.

For the SIRT1 activator screening assay, MQ water and kit reagents (SIRT1 Assay Buffer, Fluoro-Substrate Peptide, and NAD⁺) were dispensed into microtiter wells. Resveratrol (LKT Laboratories, MN, USA) and Gnetin C, prepared as previously described^11^, were applied at 10 − 1,000 µM (each in 0.1% DMSO) or vehicle control (0.1% DMSO), after which Developer and recombinant SIRT1 were added, and the reaction was incubated at room temperature. Fluorescence was measured 10 min after enzyme addition at Ex/Em = 350/460 nm using a BioTek Cytation 5 plate reader. This protocol measures the direct effects of test compounds on recombinant SIRT1 deacetylase activity under defined, NAD⁺-dependent conditions; increases in fluorescence indicate activation, whereas decreases indicate inhibition, independent of cellular signaling contexts.

### Statistical analysis

Experimental data are presented as the mean ± SEM. Statistical significance was determined using one-way ANOVA followed by Dunnett’s post-hoc test, with a significance level set at *p* < 0.05. All statistical analyses were performed using GraphPad Prism 9 (GraphPad Software, California, USA).

## Supplementary Information

Below is the link to the electronic supplementary material.

Supplementary Material 1

## Data Availability

The datasets used and/or analyzed during the current study are available from the corresponding author on reasonable request.
