# Supplementary material for: Melinjo-derived Gnetin C restores metabolic balance via dual adipose and hepatic effects in high-fat diet mice
Source: Sci Rep. 2025 Nov 25;15:41801. doi: 10.1038/s41598-025-25705-x (PMC12647683; doi:10.1038/s41598-025-25705-x)
Supplement: Supplementary file 1 — Supplementary Material 1 [file 41598_2025_25705_MOESM1_ESM.pdf]

Melinjo-derived Gnetin C restores metabolic balance via dual adipose and hepatic effects in high-fat diet mice

Tomoki Kishimoto[1,2], Aoi Nasu[1], Mai Uemura[1], Keisuke Kawano[1], Choyo Ogasawara[1], Ayami Fukuyama[1], Taisei Kawakami[1], Hirofumi Nohara[1,3], Ryunosuke Nakashima[1], Noriki Takahashi [1,3], Yukio Fujiwara[4], Tomoki Ikuta[5], Mary Ann Suico[1,6], Hirofumi Kai[1,6], Tsuyoshi Shuto[1,6,*]

Supplemental Figures

Supplemental Figure S1 : High-dose administration of Gnetin C does not cause body weight loss in WT mice

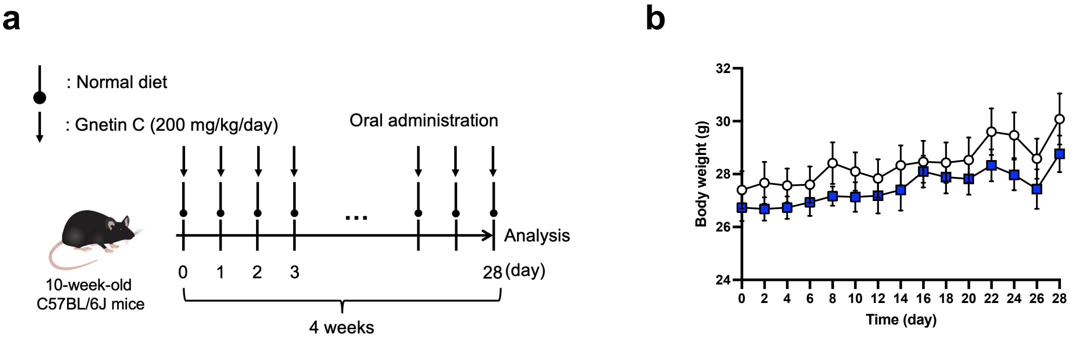

a, b: The oral gavage of GnetinC (200 mg/kg) every day for 4 weeks in WT mice. **b**: Body weight changes in WT mice treated with vehicle or GnetinC (200 mg/kg). Data are the means ± SEM; 6 mice/group.

Supplemental Figure S2 : Expression changes of APN multimerization-promoting factors

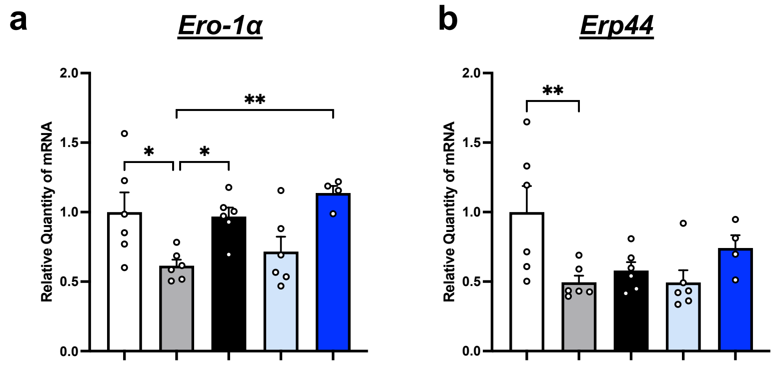

a, b: The relative quantity of mRNA levels of Ero-1α (a) and Erp44 (b) in the adipose tissue of HFD-mice treated with vehicle, MSE (1,000 mg/kg), and Gnetin C (100, 200 mg/kg). Data are the means ± SEM; 4-6 mice/group. P values were assessed by ANOVA with Dunett's test. (*$P < 0.05$, **$P < 0.01$).

Supplemental Figure S3 : Full-length gel images of Figure. 2h

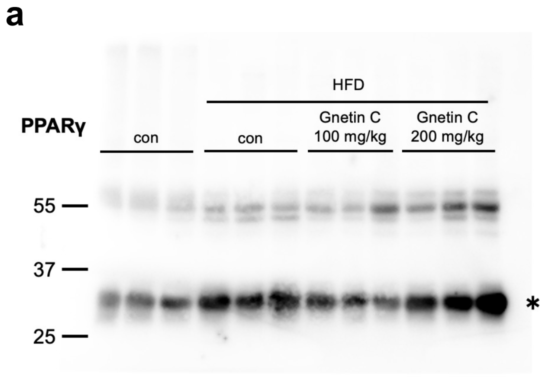

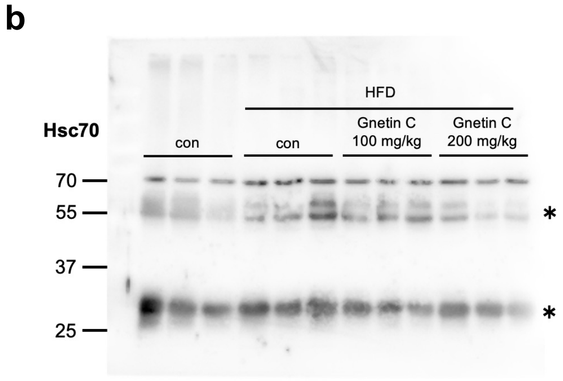

a, b: Western blotting data using anti-PPARγ (**a**) and anti-Hsc70 (**b**) antibodies. Molecular weight was indicated based on the dual color marker (Bio-Craft). * indicates non-specific band.

Supplemental Figure S4 : Full-length gel images of Figure. 4i

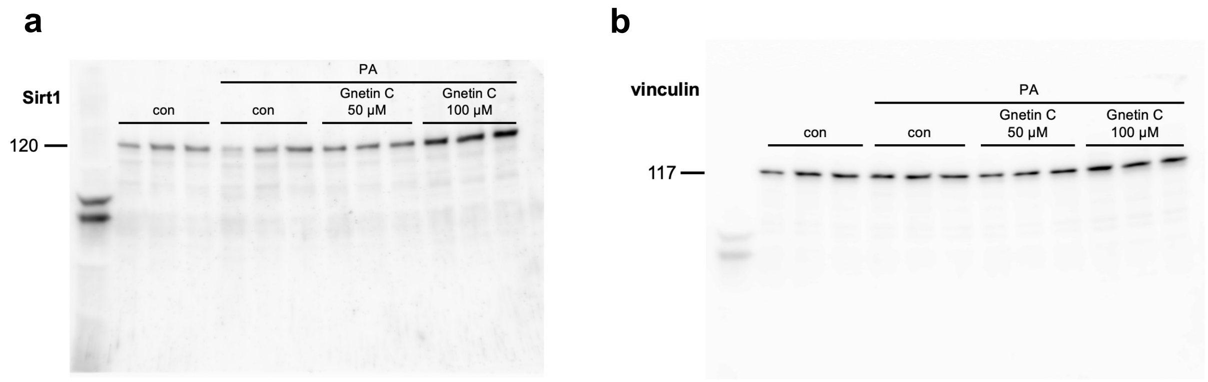

a, b: Western blotting data using anti-Sirt1 (**a**) and anti-vinculin (**b**) antibodies. Molecular weight was indicated based on the dual color marker (Bio-Craft). * indicates non-specific band.
